# PFHxS Exposure and the Risk of Non-Alcoholic Fatty Liver Disease

**DOI:** 10.3390/genes15010093

**Published:** 2024-01-13

**Authors:** Zulvikar Syambani Ulhaq, William Ka Fai Tse

**Affiliations:** 1 Laboratory of Developmental Disorders and Toxicology, Center for Promotion of International Education and Research, Faculty of Agriculture, Kyushu University, Fukuoka 819-0395, Japan; 2 Research Center for Pre-Clinical and Clinical Medicine, National Research and Innovation Agency Republic of Indonesia, Cibinong 16911, Indonesia

**Keywords:** transcriptomic, toxicities, PFAS, zebrafish, chronic liver disease

## Abstract

Perfluorohexanesulfonic acid (PFHxS) is a highly prevalent environmental pollutant, often considered to be less toxic than other poly- and perfluoroalkyl substances (PFASs). Despite its relatively lower environmental impact compared to other PFASs, several studies have suggested that exposure to PFHxS may be associated with disruptions of liver function in humans. Nevertheless, the precise pathomechanisms underlying PFHxS-induced non-alcoholic fatty liver disease (NAFLD) remain relatively unclear. Therefore, this study applied our previously published transcriptome dataset to explore the effects of PFHxS exposure on the susceptibility to NAFLD and to identify potential mechanisms responsible for PFHxS-induced NAFLD through transcriptomic analysis conducted on zebrafish embryos. Results showed that exposure to PFHxS markedly aggravated hepatic symptoms resembling NAFLD and other metabolic syndromes (MetS) in fish. Transcriptomic analysis unveiled 17 genes consistently observed in both NAFLD and insulin resistance (IR), along with an additional 28 genes identified in both the adipocytokine signaling pathway and IR. These shared genes were also found within the NAFLD dataset, suggesting that hepatic IR may play a prominent role in the development of PFHxS-induced NAFLD. In conclusion, our study suggests that environmental exposure to PFHxS could be a potential risk factor for the development of NAFLD, challenging the earlier notion of PFHxS being safer as previously claimed.

## 1. Introduction

Aligned with the escalating global rates of diabetes and obesity, there has been a substantial rise in the prevalence of NAFLD in recent years [1]. This condition has emerged as one of the most prevalent contributors to chronic liver disease on a worldwide scale [2,3], and it is estimated to impact approximately a quarter of the global population [4]. While this estimation could potentially still underestimate the occurrence of early preclinical stages, it is important to note that these stages do not always progress uniformly. This is primarily because diagnosing NAFLD requires a complex investigation, and in many cases, NAFLD has been underdiagnosed, leading to missed preventive opportunities [5]. Furthermore, even with substantial global vaccination initiatives and the success of antiviral treatments in reducing hepatocellular carcinoma (HCC) cases associated with HBV/HCV infections, there has been a surge in HCC cases tied to NAFLD [6]. Projections point towards NAFLD as an emerging cause of the HCC incidence in the upcoming years. Therefore, it is crucial to identify the risk factors linked to non-viral-related HCC, especially within high-risk populations. Moreover, by comprehensively understanding these risk factors, we can significantly enhance our capacity to detect HCC early, intervene effectively, and implement targeted prevention strategies. This proactive approach not only enhances patient outcomes but also empowers healthcare professionals to customize interventions and screening protocols for at-risk individuals, thereby reducing the burden of HCC within these specific populations.

Recently, exposure to environmental chemicals has been revealed to significantly contribute to liver disease, including NAFLD [1]. Moreover, accumulating evidence underlines the hepatotoxic effects of PFAS [6,7]. While human exposure to PFAS has been predominantly linked to contaminated food and drinking water, other potential sources include inhalation of indoor air and dust, as well as contact with various consumer products, household articles, cleaning agents, and personal care products [8,9]. The extended environmental persistence and prolonged biological half-lives of certain PFASs have resulted in detectable levels of these compounds in the blood of nearly the entire population in developed countries [10]. It is worth noting that while PFAS compounds with shorter carbon chains, like PFHxS, generally exhibit lower toxicity [11], our recent study, along with other studies, has revealed the presence of PFHxS bioaccumulation in zebrafish larvae and the *Pseudomonas* sp. [11,12]. This implies the lack of potential biological catalysts capable of fully decomposing these short-chain fluorochemicals, highlighting the concerning rise in the levels of short-chain PFASs within the environment. Together, these observations underscore the potential risks associated with the accumulation and persistence of PFASs, prompting an urgent need for a more extensive investigation into their multifaceted impact on both human health and the environment. Understanding the intricate ways in which these substances persist and accumulate, along with their potential implications for human health, ecological systems, and the broader environment, becomes increasingly imperative. Such a comprehensive exploration can guide the development of robust regulatory measures and targeted interventions to mitigate the adverse effects of these chemicals, safeguarding both human well-being and ecological balance.

Due to their persistence, bioaccumulation, and toxicity (PBT), long-chain PFASs are listed as substances controlled by the European Union (EU) [13]. In response to the demands of the electroplating industry, which encompass products featuring “non-stick” attributes and surface-tension reduction properties [8], there has been a substantial increase in the production of PFHxS as a replacement for perfluorooctane sulfonic acid (PFOS) and perfluorobutanoic acid (PFOA) [14]. Furthermore, among the most used short-chain PFASs, PFHxS stands out as a remarkably efficient surfactant due to its complete fluorination along the alkyl compound, resulting in superior physical and chemical stability [11]. While PFHxS is generally considered to be less toxic than PFOS, it shares numerous comparable physical and chemical properties with PFOS [15]. This similarity implies that PFHxS could potentially elicit biological effects similar to those of PFOS. A recent human study in the United States demonstrated that among the various PFAS compounds examined, only the serum levels of PFHxS showed a significant association with a higher risk of fatty liver disease (FLD) after adjusting for alcohol consumption levels [16]. In line, children exposed to PFHxS exhibited an increased susceptibility to the development of non-alcoholic steatohepatitis (NASH) when compared to those exposed to PFOS [17]. Despite the aforementioned findings, the possible mechanism by which PFHxS induces liver dysfunction remains unclear. Hence, in this study, we would like to uncover such a mechanism by summarizing the related publications and reanalyzing our published transcriptome data (BioProject Accession 908883) to understand the potential mechanisms underlying the development of PFHxS-induced NAFLD. Through the comprehensive investigation proposed in this study, our primary objective is to significantly contribute to advancing our understanding of the intricate impact that PFHxS exposure has on liver health. Furthermore, by delving into the complexities of PFHxS-induced effects on liver function, cellular processes, and molecular pathways, this study may illuminate novel preventive and therapeutic strategies for addressing the adverse consequences of PFHxS exposure on liver health. Thus, this study may not only unravel the underlying mechanisms of PFHxS-induced NAFLD but also identify potential biomarkers, therapeutic targets, and intervention strategies for preventing PFHxS-related liver disorders. Ultimately, this study will provide valuable knowledge essential for safeguarding liver health in the face of PFHxS exposure, thereby contributing to improved public health and well-being.

## 2. Materials and Methods

The details of the zebrafish exposure experiments and transcriptomic analysis have been outlined in our previous publication [12]. Briefly, twenty-five larvae in total were combined to form a single biological sample. The extraction of total RNA from both the control group and larvae exposed to 5 µM PFHxS (*n* = 3 for each group) was conducted using ISOGEN (Nippon Gene, Toiya-machi, Toyama, Japan) for subsequent transcriptome sequencing. The extracted RNA underwent quality assessment via the Agilent 2100 Bioanalyzer system (Agilent Technologies, Santa Clara, CA, USA), ensuring that all prepared samples had an RNA integrity number exceeding 8. Standard mRNA library construction procedures were followed, culminating in DNA nanoball synthesis. Subsequently, the samples were sequenced using the DNBSEQ platform, employing a sequencing length of PE100. To ensure data integrity, the sequence reads underwent filtration using SOAPunke (v1.5.2) with the following parameters: “-l 15 -q 0.2 -n 0.05”. This filtration process eliminated low-quality reads (those with bases scoring less than 15, constituting more than 20% of the total bases in the read), reads containing adaptor sequences, and those with high levels of N bases (>5%). The alignment of sequencing reads was accomplished using HISAT software (v2.0.4) with the specified parameters: sensitive; --no-discordant; --no-mixed -I 1 -X 1000 -p 8; rna-strandness, RF. The resulting clean reads were then mapped to the Danio rerio reference genome (Ensembl release-105) employing Bowtie2 software (v2.2.5) with the parameter set as “-q --sensitive --dpad 0 --gbar 99999999 --mp 1,1 --np 1 --score-min L,0,−0.1 -p 16 -k 200”. Subsequently, gene expression levels were computed utilizing the RSME software package (v1.2.8) with parameters set as **“**-p 8 --forward-prob 0 --paired-end”.

In this specific analysis, we identified differentially expressed genes (DEGs) in our transcriptomic dataset by applying the criteria of log2FC ≥ 1 and a Q value ≤ 0.05. Subsequently, based on DEG data, we conducted Kyoto Encyclopedia of Genes and Genomes (KEGG) disease and pathway analyses using BGI’s Dr. Tom system (http://biosys.bgi.com, accessed on 22 August 2023). Finally, the list of DEGs in each selected term were then further evaluated and a Venn diagram from the selected terms was generated using InteractiVenn [18]. A KEGG network diagram was generated based on the genes commonly shared between the two selected KEGG terms. To validate DEGs from the transcriptomic analysis, qRT-PCR was conducted using total RNA extraction from 5-dpf larvae and ISOGEN (Nippon Tech), followed by cDNA synthesis (*n* = 4). In total, 0.5 µg of RNA was reverse transcribed using Superscript VILO (Thermofisher, Waltham, MA, USA). Real-time PCR was conducted on the QuantStudio 3 Real-Time PCR system (Thermofisher) using Power SYBR^®^ Green PCR Master Mix (Thermofisher) and the primer sets listed in Table 1, as previously described [19,20,21]. Graphs and statistical analyses were conducted using GraphPad Prism 9. All data are expressed as the mean ± SEM. Statistical significance was evaluated using an unpaired Student’s *t*-test. A significance level of *p* < 0.05 was considered statistically significant. All experimental procedures on zebrafish embryos were approved by the animal ethics committee of Kyushu University, Japan (A22–103–0). A comprehensive literature search was conducted from PubMed, Scopus, and Web of Science using keywords such as “zebrafish”, “PHFxS”, “PFAS”, “NAFLD”, and “endocrine” without applying a language restriction and dated up to September, 2023. Data were then extracted as depicted in Table 2.

## 3. Results and Discussion

### 3.1. Developmental Toxicities of PFHxS across Zebrafish Studies

In this study, we selected zebrafish as our model to examine the toxicities of PFHxS. Our study, along with nine others [12,22,23,24,25,26,27,28,29,30,31], has been incorporated and is presented in Table 2. The developmental toxicities of PFHxS in zebrafish have shown varying results across different studies (Table 2). Nonetheless, our study revealed that developmental changes, particularly in relation to cranial development, started to occur when fish were exposed to 5 µM PFHxS [12]. Furthermore, it is important to highlight that hepatic lipid accumulation may have occurred as a result of hepatocyte vacuolation in the mixture of PFAS, including PFHxS [26]. Similarly to what was observed in PFOS exposure [32,33], disruptions in fatty acid β-oxidation (FAO), characterized by the upregulation of *cpt1a*, have also been reported in fish exposed to PFHxS [30,31]. This implies that PFHxS shares similarities with PFOS in terms of its impact and may potentially result in similar toxicities, even though earlier claims had promoted the notion of lower toxicities associated with PFHxS. Hence, further investigation and reassessment of the potential risks associated with PFHxS exposure is needed. Furthermore, understanding the complexity of assessing the health implications of various PFAS compounds emphasizes the significance of continually advancing our understanding to better protect human health and the environment from these potentially harmful chemicals.

In addition to the previously mentioned findings, there is also evidence of hyperglycemia in fish exposed to PFHxS [12,30,31], which may be attributed to disruptions of β-cell function [23] and the development of IR [34]. Indeed, elevated PFHxS levels have been correlated with the dysregulation of glucose metabolism in females during puberty and in obese children [35,36]. While the relationship between PFHxS exposure and MetS remains inconsistent across studies [37,38,39], multiple lines of evidence indicate that serum levels of PFAS, including PFHxS, are associated with an increased risk of MetS [38,40,41]. Consistent with this, our transcriptomic analysis reveals significant metabolic changes in PFHxS-exposed zebrafish [12], implying a potential connection between PFHxS exposure and MetS development, particularly related with components of the syndrome. Nonetheless, it is crucial to emphasize the need for further investigations involving different animal models as well as a larger sample size to strengthen and validate these findings. Such investigations are crucial not only for validating these initial observations but also for deepening our understanding of the intricate mechanisms responsible for PFHxS-induced metabolic alterations. Ultimately, these efforts will advance our ability to mitigate potential risks associated with PFAS exposure.

### 3.2. Possible Mechanism of PFHxS Induced the Development of NAFLD

Bioinformatics analysis revealed that the endocrine system, particularly in relation to the genetic aspects of obesity, ranked highest among KEGG disease term for 5 μM PFHxS-exposed fish, as outlined in Table 3. In line, among the top 12 KEGG pathways, three were closely associated with endocrine and metabolic disorders: the adipocytokine signaling pathway, IR, and NAFLD (Table 4). Notably, NAFLD emerged as the most prominent pathway, exhibiting the highest gene size among the top 12 (Table 3), implying that exposure to and bioaccumulation of PFHxS may potentially induced the development of NAFLD, possibly through the disruption of the endocrine system.

**Figure 1:**
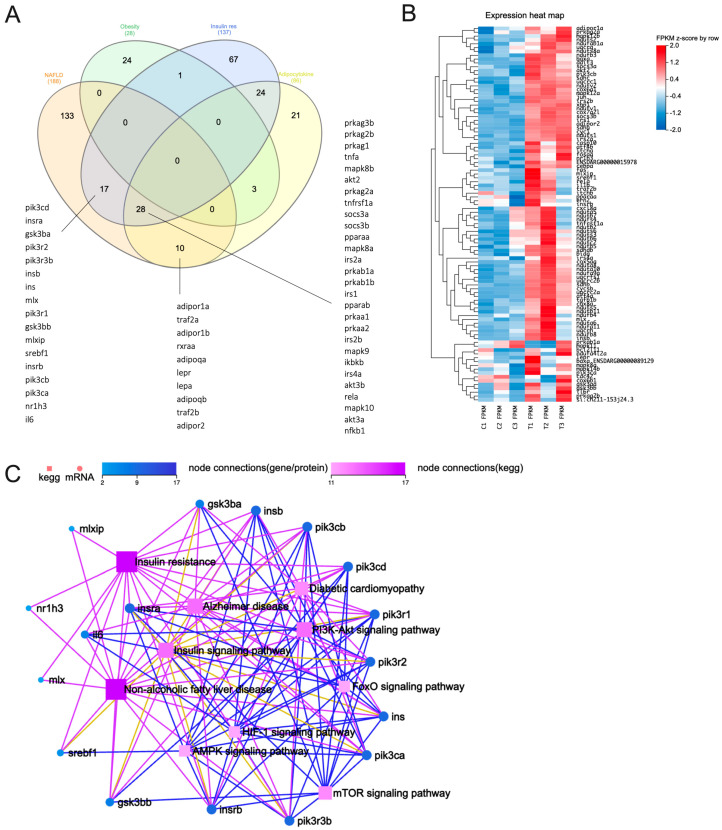
Transcriptomics analysis of 5-dpf zebrafish larvae exposed to 5 μM PFHxS. (**A**) Venn diagram illustrating enriched genes within each selected term. (**B**) The ninety-four genes identified as core enrichment genes associated with NAFLD, predominantly showing upregulation upon exposure to 5 µM PFHxS. (**C**) A KEGG network diagram based on the 17 genes consistently detected in both NAFLD and IR.

We thus then selected the aforementioned terms and assessed the genes enriched within these selected terms. For a detailed list of enriched genes within each term, please refer to Tables S1–S4. Our analysis revealed that seventeen genes were consistently found in both NAFLD and IR, while an additional twenty-eight genes were identified in both the adipocytokine signaling pathway and IR, with these shared genes also being present in the NAFLD dataset. Ten genes were found to be commonly shared between the adipocytokine signaling pathway and NAFLD. It was worth noting that none of the genes enriched in genetic obesity were identical to those associated with NAFLD (Figure 1A). Collectively, our findings support the notion that chronic liver disorders like NAFLD are strongly associated with IR. This reinforces the significance of exploring the shared genetic landscape in elucidating the complex relationship between IR and NAFLD pathogenesis. However, the intricate nature of this association warrants thorough investigation and further in-depth analyses and experimental validation.

Out of the 188 genes that were enriched in NAFLD, 94 were identified as core enrichment genes associated with NAFLD (as shown in Figure 1B, see Table S4). These genes were primarily upregulated in response to exposure to 5 µM PFHxS (Figure 1B). Furthermore, the pathway analysis conducted using the seventeen genes commonly shared between NAFLD and IR (Figure 1A) unveiled six significant signaling pathways: the phosphoinositide 3-kinase (PI3K)/Akt, insulin, mTOR, FoxO, AMPK, and HIF-1 signaling pathways. Notably, the PI3K/Akt and insulin signaling pathways exhibited a greater number of interconnected nodes (Figure 1C), indicating their potential involvement in the pathogenesis of NAFLD induced by PFHxS. While our analysis may not directly link obesity to the development of NAFLD, it is conceivable that genes associated with obesity might elicit sub-acute inflammation, which in turn may contribute to hepatic IR in NAFLD [42]. This is supported from the findings that fish exposed to a mixture of PFAS compounds containing PFHxS displayed histological alterations characterized by hepatocyte vacuolation and oxidative stress, resulting in fat infiltration into the liver [26]. Such changes could potentially indicate a disruption in insulin signaling [43]. Therefore, it seems that hepatic IR is prominently involved in the development of PFHxS-induced NAFLD, rather than extrahepatic IR.

**Figure 2:**
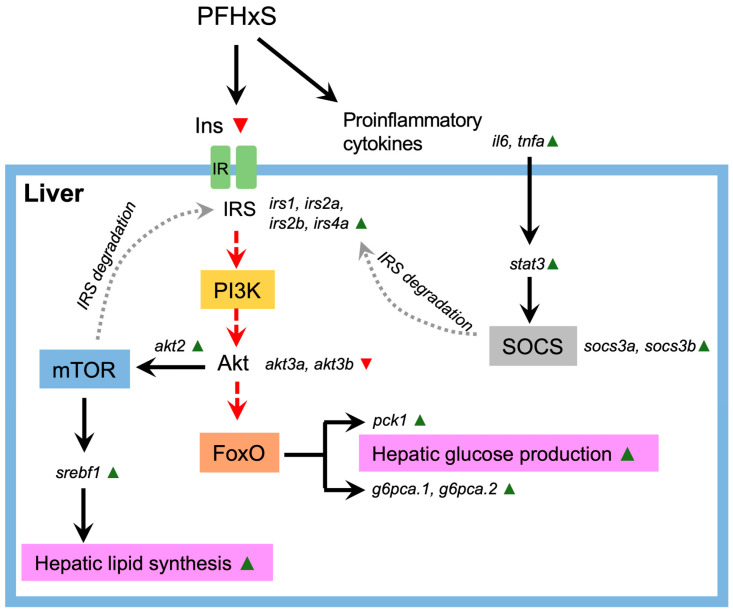
Exposure to PFHxS triggers a cascade of events leading to NAFLD. Pro-inflammatory cytokines, activated in response to PFHxS exposure, initiate the ubiquitin-mediated proteasomal degradation of insulin receptor substrates 1 and 2 (IRS1/2), essential molecules for insulin transduction, via the phosphoinositide 3-kinase (PI3K)/Akt pathway. This degradation is facilitated through the activation of the suppressor of cytokine signaling (SOCS) by the signal transducer and activator of transcription 3 (STAT3). Alternatively, the proteasomal degradation of IRS1/2 can promote mTOR signaling. The depletion of IRS1/2 disrupts Akt signaling, impairing Akt-mediated phosphorylation and leading to the further ubiquitin-mediated proteasomal degradation of forkhead box protein O (FoxO). FoxO activation subsequently upregulates gluconeogenic enzymes such as phosphoenolpyruvate carboxykinase (PCK1) and glucose-6-phosphatase (G6P), resulting in increased hepatic glucose production. Conversely, the PFHxS activation of *akt2* promotes the expression of sterol regulatory element-binding transcription factor 1 (*srebf1*, also known as sterol regulatory element-binding protein 1 (SREBP-1)), which in turn enhances lipid synthesis in the liver. Together, these mechanisms exacerbate the progression of PFHxS-induced NAFLD. Black, continuous lines: activation pathways. Red, dashed lines: inactivated pathways. Grey, dashed lines: possible stimulation/induction. Green triangle: upregulated genes. Red triangle: downregulated genes.

Although PI3K/Akt and its downstream target, such as mTOR and FoxO, are well established as targets of insulin signaling [42,44], it is possible that these pathways act independently in promoting hepatocyte steatosis in PFHxS-induced NAFLD through differential mechanisms [45]. Additionally, it appears that other factors, such as the overstimulation of inflammatory cytokines within the liver due to PFHxS exposure, may further contribute to the increased lipid accumulation in hepatocytes. While PFHxS suppressed the expression of insulin (*ins*), our findings indicated an upregulation of insulin receptor substrates (IRS) in our case (Figure 2), possibly as a compensatory response to the disrupted insulin signaling. However, a study suggests that the excessive influx of pro-inflammatory cytokines into lipid and Kupffer cells stimulates the degradation of IRS1/2, leading to IR and an inhibition of the PI3K/Akt pathway [42]. PFHxS indeed induced the synthesis of the suppressor of cytokine signaling (SOCS), likely through the activation of a signal transducer and the activator of transcription 3 (STAT3) mediated by TNF-α and IL-6. (Figure 2). In line with the upregulation of *akt2* by PFHxS, the suppression of PI3K interaction with IRS can also result in IRS1 degradation through the mTOR complex (Figure 2) [46,47], highlighting the significance of the interplay between the insulin and PI3K/Akt pathways in maintaining normal lipid metabolism. Due to hepatic IR, the insulin signaling cascade through Akt/FoxO is disrupted (characterized by elevated expression of phosphoenolpyruvate carboxykinase 1 (*pck1*) and glucose-6-phosphatase catalytic subunit 1a, tandem duplicate 1/2 (*g6pca.1*, *g6pca.2*)), whereas the mTOR/sterol regulatory element binding protein 1 (SREBP1, encoded by *srebf1*) pathway remains active, leading to increased hepatocellular glucose production and lipogenesis [42,44,45], further exacerbating the clinical progression of PFHxS-induced NAFLD (Figure 2). This intricate molecular interplay underscores the multifaceted nature of PFHxS-induced NAFLD pathogenesis, emphasizing the pivotal role played by disruptions in insulin signaling pathways and their downstream effects on lipid metabolism. Understanding these complex interactions holds promise for developing targeted interventions aimed at mitigating the adverse effects of PFHxS on hepatic health.

**Figure 3:**
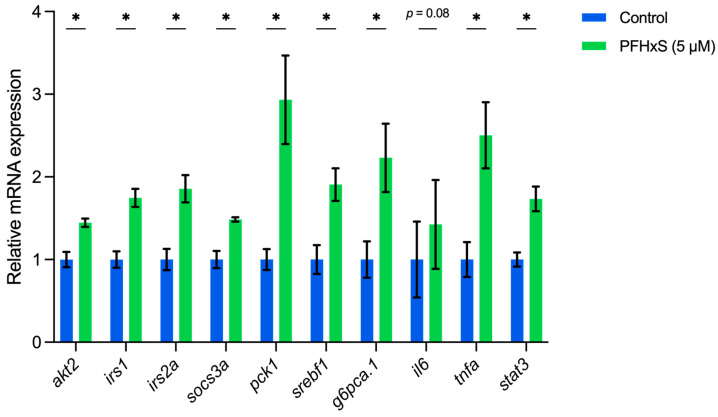
The mRNA expression levels of 10 selected genes (*akt2*, *irs1*, *irs2a*, *socs31*, *pck1*, *srebf1*, *g6pca.1*, *il6*, *tnfa*, and stat3) evaluated in PFHxS-exposed fish and compared to the control group at 5-dpf. Data are expressed as mean ± SEM. * indicates *p* < 0.05.

Utilizing qRT-PCR analysis, we conducted a validation of the aforementioned signaling pathways (as depicted in Figure 2) by measuring the mRNA expression levels of 10 specific genes, as detailed in Table 1. Remarkably, within PFHxS-exposed fish, the expression of these genes exhibited a substantial upregulation when compared to that of the control group, with the exception of *il6* (as shown in Figure 3). Nonetheless, the expression patterns of *il6*, along with *tnfa* and *stat3*, exhibited a tendency toward elevation in comparison to controls, albeit without reaching statistical significance (*p* = 0.08), suggesting potential excessive inflammatory responses in PFHxS-exposed fish. While the association between PFAS and IR varies among studies [48,49], some studies have indicated positive associations between multiple PFAS and the development of type 2 diabetes or IR [50]. Nevertheless, our study emphasizes the significant contribution of hepatic IR to the advancement of hepatic steatosis in PFHxS-induced NAFLD. Altogether, this observation offers a critical insight into the complex interplay between PFHxS exposure, IR, and the advancement of NAFLD, shedding light on a potential mechanistic link that warrants further exploration in understanding the pathogenesis of NAFLD triggered by PFHxS exposure.

## 4. Conclusions

In conclusion, the findings of this study suggest that environmental exposure to PFHxS may be a potential risk factor for the development of NAFLD. Furthermore, hepatic IR induced by PFHxS seems to play a significant role in the pathogenesis of NAFLD, and there could be a synergistic effect between PFHxS and modifiable risk factors, such as obesity, in the context of NAFLD. Despite validation through qRT-PCR analysis, pathways such as Akt activation, STAT3 activation, IRS degradation, and mTOR rely on protein-level alterations, including changes in protein stability or phosphorylation. Therefore, additional validation through the Western blot technique is necessary. Finally, we anticipate that the insights derived from this study will serve as a catalyst, prompting further inquiry into the intricate pathogenesis of NAFLD. Specifically, the examination of NAFLD in the context of environmental pollutants such as PFHxS encourages a deeper exploration that warrants substantial attention and subsequent investigation.

## Figures and Tables

**Table 1:** Primer list for qRT-PCR experiments.

No	Gene	Forward	Reverse
1	*akt2*	ACGCGAGATCGACTGTGTTT	GCTGAAACGATTTCTGCCCC
2	*irs1*	TGACTGCCTCTTTCCACGTC	CTTCGAAAGTCACAGGGGCT
3	*irs2a*	TTCGACGGCCTCATTTCACA	GCATGTTCTGTTGTTAAAAGCTCTG
4	*socs3a*	GCTTACGTTTTTGGGCCTGG	GCAAGAATGGCGCTTCAACA
5	*pck1*	GAGCTCTTCAGGGTCTCGC	AGATTAACGTGTGTGTTGCGT
6	*srebf1*	ACTCTGAAACCGGACGTGAC	TACGGTTGATGGGCAGCTTT
7	*g6pca.1*	ACACAACGGGTGGCTACAAA	TTTGCTTCGATGAACTTGGGT
8	*stat3*	ACAGTGAGCTGCTTGGGAAC	TATCCGAGACTGTGGAGGCT
9	*il6*	CCTCAGTCCTGGTGAACGAC	TGCGAGTCCATGCGGATTTA
10	*tnfa*	TTGCCTTTACCGCTGGTGAT	CCTGGGTCTTATGGAGCGTG
11	*β-actin*	TTGACAACGGCTCCGGTATG	TCCCATGCCAACCATCACTC

**Table 2:** The effect of PFHxS exposure in different zebrafish studies.

Study	PFHxS Concentration	Major Findings		
		Developmental and Behavioral Toxicities	Liver Function	Endocrine/Metabolic Problems
Ulhaq and Tse, 2023 [11]; Ulhaq et al., 2023 [22]	0.1–10 µM	Developmental toxicity can be observed to have started at 5 µM	Lipid accumulation was not assessed in the liver; however, it was observed in the GIT.	Hyperglycemia, hyperactivation of glucose uptake,
Annunziato et al., 2020 [23]; Annunziato et al., 2019 [24]	100–1000 µM 0.011–0.22 ng/mL	LC_50_ = 340 µM PFHxS up to 22.5 mg/L did not show any morphological defect	Aqueous film-forming foam (AFF) exposure reduced liver size	AFF leads to the disruption of β cells, resulting in their fragmentation, and negatively impacting the growth and development of the pancreas
Gaballah et al., 2020 [25]	0.4–80 µM	EC_50_ = 92.7 µM Hyperactivity at 14–25.1 µM	NA	NA
Huang et al., 2022 [26]	1–100 ng/mL	NA	PFHxS tightly bind to the active pocket of ZSA and ZL-FABP, lipid accumulation in the liver possibly due to hepatocyte vacuolation	NA
Menger et al., 2020 [27]	12–60 µM	Reduction in swimming activity in dark environments and increased burst swimming activity	NA	NA
Phelps et al., 2023 [28]	0.03–80 µM	AC_50_ = 28.63 µM Suppression of respiratory burst	NA	NA
Vogs et al., 2019 [29]	0.4–330 µM	EC_50_ = 84.5 µM	NA	NA
Xu et al., 2023 [30]; Xu et al., 2022 [31]	0.3–10 µM	NA	Dysregulation of FAO	A glucose metabolism defect marked by the inhibition of the hydrolysis of large-molecular sugar

FAO, fatty acid β-oxidation; NA, not available; GIT, gastrointestinal tract; LC_50_, median lethal dose; EC_50_, half-maximal response dose; AC_50_, the concentration for half-maximal activity derived from the Hill equation model; ZSA, zebrafish serum albumin; ZL-FABP, zebrafish liver fatty acid-binding protein.

**Table 3:** KEGG disease term in PFHxS exposed fish.

No.	Category	KEGG Disease Term	Size	FDR q-Value
1	H02106	Genetic obesity	28	0.000
2	H00891	Combined oxidative phosphorylation deficiency	64	0.000
3	H00069	Glycogen storage disease	52	0.005
4	H00292	Hypertrophic cardiomyopathy	64	0.006
5	H01762	Muscle glycogen storage disease	38	0.008

**Table 4:** KEGG pathways in PFHxS exposed fish.

No.	Category	KEGG Pathway Term	KEGG Pathway Term Level 1	KEGG Pathway Term Level 2	Size	FDR q-Value
1	4140	Autophagy-animal	Cellular Processes	Transport and catabolism	184	0.000
2	4920	Adipocytokine signaling pathway	Organismal Systems	Endocrine system	86	0.000
3	4657	IL-17 signaling pathway	Organismal Systems	Immune system	83	0.000
4	4136	Autophagy-other	Cellular Processes	Transport and catabolism	31	0.000
5	4137	Mitophagy-animal	Cellular Processes	Transport and catabolism	92	0.000
6	1230	Biosynthesis of amino acids	Metabolism	Global and overview maps	87	0.00001
7	4931	Insulin resistance	Human Diseases	Endocrine and metabolic disease	137	0.00005
8	4620	Toll-like receptor signaling pathway	Organismal Systems	Immune system	97	0.00004
9	970	Aminoacyl-tRNA biosynthesis	Genetic Information Processing	Translation	43	0.00039
10	4621	NOD-like receptor signaling pathway	Organismal Systems	Immune system	155	0.00047
11	20	Citrate cycle (TCA cycle)	Metabolism	Carbohydrate metabolism	34	0.00043
12	4932	Non-alcoholic fatty liver disease	Human Diseases	Endocrine and metabolic disease	188	0.00039

## Data Availability

The transcriptomic data has been deposited under the reference Bio Project Accession 908883, accessible at https://www.ncbi.nlm.nih.gov/bioproject/?term=908883 (accessed on 22 August 2023).

## Supplementary Materials

The following supporting information can be downloaded at https://www.mdpi.com/article/10.3390/genes15010093/s1: Table S1. List of enriched genes associated with genetic obesity in PFHxS-exposed fish; Table S2. List of enriched genes associated with adipocytokine signaling pathway in PFHxS-exposed fish; Table S3. List of enriched genes associated with insulin resistance in PFHxS-exposed fish; Table S4. List of enriched genes associated with non-alcoholic fatty liver disease in PFHxS-exposed fish.
